# 

**Published:** 1882-01

**Authors:** 


					﻿VOLJJU
JANUARY, 1882.
NO. 1 ’
IPHE
HOMEOPATHIC pHY^lClAfJ,
A MONTHLY JOURNAL OF MEDICAL SCIENCE.
“ J/a/ywa est veritas, et proevalebity
E. CT. LEE, JVC. ZD.
EDITOR. >	■
CONTENTS:
(See inside of Cover.)
CONTRIBUTORS:
Drs. H. C. Allen, T. F. Allen, Edward Bayard, J. B. Bell, E. W. Berridge,
Geo. H- Clark, A. C. Cowperthwaite, A,d. Fellger, Wm. J. Guernsey,
W. A. Hawley, John Hall, W. M. James, W. H. Jenny, Ad. Lippe,
- i •£• Lippe, Malcolm MacFarlan, Thos. Moore, C. F. Nichols,
C. Pearson, T. F. Pomeroy, Leila A. BenDell, 0. Carle-
ton Smith, P. P. Wells, Wm. P. Wesselhoeft,
-David Wilson, (London), T. P. Wilson,
and many others
Philadelphia 4
No. 2109 CHESTNUT STREET.
L O N D O 3>T.
ALFRED HEATH & CO., Sole Agents kor Great Britain,
114 Ebury Street, Eaton Square.
Yearly. Subscription, $2.00, invariably in advance.
Entered at the Philadelphia Post-Office, as Second-Class Mail Matter.
				

